# LIGHT: A Church-Based Curriculum for Training African American Lay Health Workers to Support Advance Care Planning and End-of-Life Decision-Making

**DOI:** 10.1089/heq.2020.0042

**Published:** 2020-12-30

**Authors:** Jerry C. Johnson, Tara Hayden, Lynne Allen Taylor, Arthur Gilbert, Marshall Paul Hughes Mitchell

**Affiliations:** ^1^ Perelman School of Medicine, University of Pennsylvania, Philadelphia, Pennsylvania, USA.; ^2^ Wissahickon Hospice, Bala Cynwyd, Pennsylvania, USA.; ^3^ Salem Baptist Church, Roslyn, Pennsylvania, USA.

**Keywords:** African American Church, palliative care, hospice, lay health workers, health equity, curriculum

## Abstract

**Purpose:** African Americans with life-limiting illnesses experience significant health inequities. Lay health workers (LHWs) may help overcome existing challenges of communicating with African Americans about advance care planning (ACP) and end-of-life decision-making. Church-based LHWs have some advantages over other LHWs but no curriculum exists to fully prepare them. This article describes the development, content, format, and implementation of a curriculum designed to meet this need.

**Methods:** We created a church-based curriculum to train African American, LHWs as communications-facilitators who can support persons with life-limiting illnesses, not only with ACP but also with issues that arise as illnesses progress. Learners are church members whom we call comfort care supporters. The curriculum organizes the LHW interactions with clients by the mnemonic LIGHT: Listening, Identifying, Guiding, Helping, and Translating.

**Results:** The final curriculum consists of three parts: (1) a 26-h classroom component delivered in nine modules organized around eight themes: meaning and prognosis of a life-limiting illness, spirituality and the meaning of death, understanding the dying process, major decisions and choices, goals of care, end-of-life services, and resources, intrafamily communication, and role and activities of the LHW; (2) a visit component; and (3) experiential, case-based discussions during monthly meetings.

**Conclusions:** LHWs may improve quality of care and thus reduce health inequities at the end-of-life. Preparing LHWs for conversations about ACP is necessary but insufficient. This curriculum also prepares LHWs to attend to the spiritual needs of clients and to support clients with their other needs as their illness progresses.

## Introduction

The care needs of persons with life-limiting illnesses has outpaced the ability of the health care workforce to provide care, and among those with life-limiting illnesses who are approaching the end-of-life, older African Americans experience significant health inequities.^1–5^ Compared with whites, African Americans with life-limiting illnesses are less likely to participate in advance care planning (ACP), or receive palliative care or hospice services, although these services have been demonstrated to improve quality of care, and African Americans who receive hospice care give it high-quality ratings.^5–12^ Consequently, African Americans are more likely to experience unmet needs for symptom management (pain and dyspnea), insufficient emotional support, absent or problematic physician communication, and lower overall satisfaction with care at the end of life.^6,7^ Informal, community, or lay health workers (LHWs) have been proposed as part of the solution to reducing these health inequities.^2^

Lay health workers working with persons with life-limiting illnesses have had variable roles including ACP, symptom management, assisting patients navigate health systems, and providing tangible assistance (e.g., household chores).^13–17^ The ability to communicate effectively is a critical determinant of the success of LHWs in most of these roles.^6,13,14,18^ When caring for African Americans with life-limiting illnesses, LHWs often face significant communications barriers. For many African Americans, conversations with health professionals and within families about death and dying only occur once death is pending; thus compressing the ACP period.^19^ Patients and families may perceive palliative and hospice care as inferior to curative care, or may view them as contrary to their faith beliefs.^1,13^ These communications barriers are exacerbated as illnesses progress and death is anticipated. During this period, patients and families experience significant emotional stress and intrafamily conflicts that are not present during the early stages of a life-limiting illness.^20–23^ Therefore, LHWs must be prepared to not only assist persons with ACP, they must “prepare people and their surrogates to be able to make decisions in real time” as illnesses progress.^24^ Instead, LHWs caring for persons with life-limiting illnesses have sometimes felt unprepared, expressing “a lack of confidence to meet the overwhelming needs of people with serious illness.”^17,18,23^

Spiritual and religious concerns should be anticipated when supporting persons with life-limiting illnesses. Interviews of patients with serious or life-limiting illnesses revealed that “attending to spiritual and religious concerns” was the most important of seven domains that influence preferences for care at the end of life, and the significance of this domain was most strong among African Americans.^14,25^ Reflecting on the challenge of communicating with patients served by care coordinator assistants (the term for LHWs in a home care program), one author concluded that it may be essential that “care coordinator assistants or trained persons within the elder's natural community (perhaps members of their religious congregation) be present to these welcomed but difficult conversations” about ACP.^14^ The African American Church presents a venue to respond to this need.^26^ As members of the spiritual family of the persons they visit, African American, church-based LHWs can offer spiritual support while also communicating about the range of issues relevant to ACP and end-of-life decision-making.^1,2,26,27^ In addition, when LHWs are linked to their church community, the imprimatur of the church creates a receptive environment in which the LHWs can conduct their work.

African American Church members have expressed interest in education and training about communication with persons with life-limiting illnesses.^26,27^ However, no curriculum exists to train African American, church-based LHWs for the primary role of communications-facilitators, serving persons who have reached the stage of a life-limiting illness, and such training presents some unique challenges. First, such an undertaking should be buttressed by church partnerships, in contrast to partnering with church members as individuals. This undertaking requires an engagement process that adheres to the principles and processes of partnering with African American Church.^26^ Second, the training must be feasible for part-time, LHWs. Third, it must be culturally appropriate and aligned with traditional African American Church norms.^26,28^ We undertook the task of creating such a curriculum and in this article we aim to describe (1) the conceptual framework of the curriculum, (2) its development, content, format, and implementation, and (3) the materials and resources required to deliver the training.

## Methods

### Conceptual framework of the curriculum

Our goal was to educate and train a cadre of African American Church members to visit fellow congregants with life-limiting illnesses, serving as a communications bridge between health professionals and clients and also as a bridge between family members. We refer to the persons visited by our LHWs as clients instead of patients because, unlike some of the other LHW programs that we reference, our LHWs do not have the professional, legal, and ethical relationship with the persons they serve that is implied by the term “patients.” Our approach was supported by social cognitive theory, which posits that learning occurs in a social context (in this case, the church) with a dynamic and reciprocal interaction of the person, environment, and behavior.^29^ The development strategy of the curriculum adhered to the steps required to develop a curriculum as a “planned educational experience” consisting of the following elements: (1) problem identification, (2) needs assessment, (3) goals and objectives, (4) implementation, (5) evaluation strategy, and (6) dissemination.^30^ The problem that we identified and that our curriculum addressed was miscommunication within the African American community about ACP, palliative and hospice care, and end-of-life decision-making, a problem identified in the literature and in our discussions with church members and leaders.^20,26,27,31–33^ The need for training is derived from focus group and other meetings with church leaders in which church members lamented the lack of education and training.^27^ A pretraining survey of the goals of our LHW learners revealed needs in two general areas: “to be more effective with church members during their time of illness,” and “to gain knowledge about caring and supporting persons with life-threatening illnesses and those at the end-of-life.”

The specific objectives of the curriculum were to: (1) impart knowledge about the dying process, the distinction between goals of care and processes of care, the meaning of ACP, palliative and hospice care, and other relevant topics, and the challenges of intrafamily communication; (2) impart skills—listening, identifying intrafamily conflicts and gaps in understanding, guiding persons to goals of care discussions, helping clients understand relevant aspects of palliative and hospice care and end-of-life decision-making, and translating health information in plain language; and (3) influence attitudes of the learners about ACP, palliative and hospice care, and the relation of faith beliefs to end-of-life decision-making. The principles of community-based participatory research and successful church-based health promotion activities provided the framework for the collaborative processes necessary for successful implementation.^26,34^ The leadership of four churches endorsed the curriculum, advocated for it throughout the process, and provided guidance and feedback about its format, content, and delivery.^26^ The evaluation and dissemination required of this curriculum are beyond the scope of this article with the exception of describing the elements of the evaluation.

## Results

### Content and format of the curriculum

We created a curriculum model that responds to the combination of distrust, misinformation, culture and faith-based beliefs, and family dynamics that lead to miscommunication (client or family with health professionals and intrafamily members) with African Americans during the period in which they experience a life-limiting illness. During this stage of illness, LHWs may experience “anxiety and fear” when trying to support persons.^17,23,27,33,35^ The final curriculum comprised three parts: classroom training, field training (visits to clients with life-limiting illnesses and their family or caregivers), and experiential, case-based discussions during monthly meetings.

We refer to our LHWs as comfort care supporters (CCSs) rather than as “navigators,” a term used to label some LHWs charged with facilitating ACP or supporting persons with serious and life-limiting illnesses.^15,16,21,22^ The navigator term grew out of programs in which LHWs were charged with advocating and intervening on behalf of patients in health systems, often obtaining or scheduling diagnostic, treatment, or social services for patients with cancer: hence the term navigator.^36–38^ However, the personal and sensitive nature of health care decisions during the death and dying period places nonfamily members (in this case, the CCSs) at risk of provoking an adverse emotional response or stimulating intrafamily discord when intervening or interjecting on behalf of clients or on behalf of one family member rather than another. For example, as part of their training, we advise CCSs not to schedule a hospice appointment or call or speak with a hospice team member on behalf of a client, even if asked. In contrast to advocacy (navigation), the CCSs comfort and support clients by listening and attending to faith beliefs and general concerns, and by preparing clients and family members to communicate effectively on their own behalf (whether with health professionals or with other family members). Instead of functioning as advocates or navigators, our CCSs empower and support the client and family as they (the client and family) enhance their capacity to function as self-advocates or self-navigators.

### Classroom curriculum

The final classroom component of the curriculum encompassed nine modules organized around eight themes (Table 1). These themes, based on the findings of our focus groups, the needs expressed in the literature, and the needs voiced by the CCSs in a pretraining survey, were presented in modular form using an interactive process of small group discussions using fact-based presentations, storytelling by teachers and learners, group exercises, and role plays. The first module established a bond among all participants (by sharing stories), established the expectations of the CCSs, and explained the meaning of a life-limiting illness. Because of a reluctance to acknowledge death as a likely consequence of a life-limiting illness and a tendency of some African Americans to view palliative and hospice care as contrary to their faith beliefs, the second module set the stage for all subsequent modules by focusing on the meaning and acceptance of death and its relation to faith beliefs.^26,27^ Modules 3–7 addressed common issues relevant to death and dying and end-of-life care and were presented in an order designed to maintain a logical flow. Modules 8–9 described the CCS's role, using cases to role play and review all content presented in the first seven modules. In addition to the emphasis on faith in Module 2, the significance of faith beliefs was diffused throughout the classroom training. Both the minister who co-led one of the modules and the learners frequently referenced the relation of faith beliefs to end-of-life decision-making and caregiving for the sick. The binder used for training referenced a biblical text at the beginning and end of each module that supported the theme of the module.

**Table 1.** Lay Health Worker Classroom Training

Purpose and theme	Time and module leaders	Emphasis	Impact
Day 1	3 h		
Module 1: pretests, orientation and meaning of a life-limiting illness	Geriatric Physician and Health Educator	Introductions, expectations, roles, prior experiences of learners; meaning and prognosis of a life-limiting illness	Knowledge and attitudes
Day 2	3 h		
Module 2: spirituality and the meaning of death	Health Educator and Chaplain/Minister	Reemphasis of expectations and roles; acceptance of death and how foregoing life sustaining treatment attempts can be in concert with spiritual beliefs	Attitudes
Day 3	8.5 h		
Module 3: understanding the dying process	Geriatric Physician and Health Educator	Appearance of the dying process; symptoms: physical, mental, emotional, and spiritual	Knowledge
Module 4: major key decisions and choices	Health Educator and Geriatric Physician	Major decisions and choices: for example, advance directives, resuscitations, feeding tubes, and others	Knowledge and attitudes
Module 5: goals of care	Geriatric Physician	Distinction between goals of care versus processes of care (diagnostic or treatment)	Knowledge
Module 6: understanding end-of-life care services and resources	Geriatric Physician and Health Educator	Advance directives, palliative and hospice care; benefits and processes; surrogate preparation	Knowledge and attitudes
Day 4	8.5 h		
Module 7: understanding family dynamics and intra-family communications	Chaplain/Minister and Geriatric Physician	Listening skills, family dynamics and how they impact decisions; respect for patient's wishes as the centerpiece of family decisions	Knowledge and skills
Module 8: the role of the comfort care supporter	Full Team	Review of the LIGHT tasks, communication skills, and ethical boundaries	Knowledge and skills
Module 9: role play: examples in action	Full Team	Skill building: case studies and opportunity to put knowledge acquired into action through role plays	Skills
Day 5	3 h 2 weeks after day 4		
Curriculum review and follow-up			
Debriefing, post-tests, and evaluation of training program	Geriatric Physician and Health Educator	Curriculum review; question and answer session	Knowledge, attitudes, and skills

#### Classroom resources and training materials

The classroom training was delivered by an academic team of three members: geriatric medicine physician, hospice chaplain and minister, and a health educator. Each team member led or co-led one of the training modules. The classroom sessions were supported by a binder organized into key (factual information, messages, and exercises) and supplemental information linked to each module. The supplemental material contained information that elaborated on the factual information and contained tips for family caregivers, a glossary of key terms, a list of regional hospice programs, other resource materials, and a reference list.

### Visit curriculum and monthly meetings

The visit experience was a critical component of the training experience. Persons with a life-limiting illness were identified by a systematic process. First, each church identified persons with potential life-limiting illnesses using traditional church venues (sick lists and word of mouth from ministers, other church leaders, church ministries, or congregants). Next, persons with a potential life-limiting illness were contacted by a church liaison, trained by the academic team, who screened the client for criteria of a life-limiting illness based on self-report of diagnoses, and more important, based on recent onset of weight loss, loss of appetite, and functional status deficits. The liaison referred the information to the geriatric medicine physician on the academic team for confirmation of a life-limiting illness. A CCS volunteered to visit the client in their home, hospital, or nursing home and to communicate by phone when face-to-face visits were unfeasible. This CCS reviewed the case by phone or email with the educator and the physician member of the team to arrive at a consensus about the most important client and family needs. Before visits, CCSs were reminded to focus their visit activities by the mnemonic, LIGHT (Table 2), which we chose to emphasize the need for CCSs to illuminate the relevant issues by applying their communications skills. CCSs were encouraged to call members of the academic team at any time to receive support.

**Table 2.** The LIGHT Mnemonic for Focusing Lay Health Worker Interactions

	Interactions encompassed by L-I-G-H-T	Curriculum link
Listen	**L**isten consistently and patiently	All modules
Identify	**I**dentify intrafamily conflicts (goals of care vs. treatment choices; patient's preferences vs. family caregiver's preferences); need for surrogate education; and gaps in understanding the illness, its prognosis, and whether there is a need for more dialog with health professionals	Modules 1,5,7,8,9
Guide	**G**uide patient and family to goals of care discussions and resources relevant to end-of-life care	Modules 4,5,6,7,8,9
Help	**H**elp patient and family understand the dying process (physical, mental, emotional, and spiritual manifestations), the benefits of PCH, and the concordance of PCH with faith beliefs	Modules 2,3,6,8,9
Translate	**T**ranslate the meaning of common terms (such as do not resuscitate, advance care directives, feeding tubes, palliative care, hospice) in plain language	Modules 6,8,9

PCH, palliative care and hospice.

To enrich the learning process, CCSs attended monthly meetings of 2 h anchored by case discussions of clients visited by the CCSs. These sessions, attended by the CCSs of each church community and the academic team, provided reinforcement of knowledge presented during the classroom sessions, peer advice about communicating with clients and families, and emotional support to alleviate the stress that CCSs sometimes experienced as a consequence of visits.

#### Visit training materials

To standardize the visits, we created visit-training materials: (1) a visit guide including tips for starting the visit (introducing oneself), stating the purpose of the visit, prayer, and asking about the illness experience of the client and caregiver, (2) a checklist of key issues organized by the LIGHT mnemonic, (3) sample prompt questions, and (4) supplementary materials. All components of the project were approved by The Institutional Review Board of the University of Pennsylvania (approval number 820859).

### Evaluation

We developed several instruments to collect the data necessary to evaluate the classroom and visit components of the curriculum: (1) Profile of the CCSs (self-reported attributes including demographics and occupation), (2) Knowledge Assessment Instrument, (3) Palliative Care and Hospice Beliefs and Attitude Assessment Form, (4) CCS Assessment of the Classroom training, (5) CCS Visit Report Form: a self-report checklist of the type of support delivered during the visits, and self-report of perceptions of the benefits and challenges of the visit. The structure of the evaluation plan and the results of the evaluation are beyond the scope of this article.

### Pilot-testing, modifications, and lessons learned

We piloted the classroom training in one of the churches and arrived at the final curriculum through an iterative process of obtaining feedback and testing modifications in the other three churches. In response to this feedback, we took several steps. We changed the order of the module topics to improve the flow of information. The major change was to present Module 5 (goals of care) after presenting Module 3 (the dying process) and Module 4 (major decisions or choices). We used this order because we found that learners needed to know the symptoms and signs (physical, mental, and emotional) associated with the dying process and the meaning, risks, and benefits of the choices (e.g., feeding tube or resuscitation) that often occur during the dying process before they could understand the difference between treatment choices and goals of care, and how treatment choices can contradict goals of care. We included discussions about ethics and privacy in each module. We disengaged the required reading from the supplemental material, and created more time for role play exercises. The final classroom component consisted of a 26-h intensive training experience: a 3-h orientation session (Module 1), followed 1 week later by eight modules delivered over 2.5 consecutive days (20 h), and a 3-h debriefing session 2 weeks later.

Discussions at the monthly meetings led to modifications in the content of the discussions and in the resources that we developed to assist the CCSs during visits. After the classroom training, CCSs did not adequately understand the symptoms and trajectories of common life-limiting illnesses such as congestive heart failure, stroke, and dementia. Some of the CCSs remained unclear about the meaning of goals of care and the benefits of palliative and hospice care. The visits created some anxiety for the learners about their performance, about how they should respond to requests for tangible support, and about uncertainty as to whether the client or family member was the priority for support. In response to these needs, we took several steps. We devoted more time during the monthly meetings than originally anticipated to review topics that were introduced in the classroom modules. We reminded the CCSs to use the LIGHT mnemonic as a tool to focus visit activities, to rely on alternative resources within the church for tangible support (such as transportation or housekeeping), to attend to the needs of the family caregiver as well as the client, and to identify content areas where clients and family need to elicit more information from a health professional. Last, we created a short visit guide containing key questions that could be used stimulate conversations about relevant topics.

## Discussion

This article describes the development, content, and format of a curriculum to partner with the African American Church to train LHWs, whom we call CCSs, to support persons with a life-limiting illness. The curriculum fills a void in the current LHW literature. In addition to preparing CCSs to engage in ACP, which is the most prevalent role of LHWs in other comparable programs, this curriculum prepares CCSs to support clients as needs arise in real time and to attend to the spiritual needs of clients. In this manner, the curriculum prepares CCSs to empower patients and family members to become informed, effective advocates for health care consistent with their goals, values, and faith beliefs. An active collaboration with church partners who served as advocates and co-leaders of our project was fundamental to the successful design and implementation of the curriculum.^26,27^ The curriculum prepared CCSs to undertake a set of activities characterized by the mnemonic LIGHT: **L**istening; **I**dentifying intrafamily dynamics and conflicts, and gaps in understanding the illness prognosis; **G**uiding persons to goals of care discussions and resources; **H**elping persons understand the dying process, the meaning of palliative and hospice care and the concordance of palliative care and hospice with faith beliefs; and **T**ranslating medical terms into plain language. The final curriculum consisted of a classroom component containing nine modules delivered over 26 h and a visit component anchored by monthly case discussions.

In the few other programs that employed LHWs to support African Americans with ACP and end-of-life decision-making, the persons served by the LHWs and the content and format of their training curricula had some similarities and differences with our curriculum.^13,14,17,21,39^ In these other programs, clients or patients were labeled by diagnoses such as “cancer” or “serious illness.” Most often, the stage of illness of these patients in relation to likelihood of death was not specified. In contrast, our curriculum targets persons who have reached the stage of illness where health professionals have predicted an inexorable decline in health and clients self-report diminutions in their functional status. The content and format of the training used in other programs included variable combinations of workshops, simulation sessions, electronic health record decision-support tools, case conferences, standardized navigation trainer courses, and virtual classrooms.^18^ The print resources used in our curriculum are less robust than the range of media approaches used in these other curricula. However, we compensate for that deficiency by our significant use of experiential learning, which is consistent with the LHW preference for “experiential learning using real case examples and role-playing over computer-based e-learning.”^18^

Lay health workers in other programs have felt unprepared for the complexities of ACP conversations and the stress of supporting persons with other aspects of end-of-life decision-making.^17,23^ The three LHW programs most similar to ours that served African Americans combined some combination of classroom, on-line training, field training, and case discussions to prepare the LHWs. In one program, “lay health advisors,” who were part-time volunteers, were recruited through African American Churches to undertake 1-day-long training session followed by monthly meetings. The goal of these lay advisors was “communicating with, advising, and supporting” persons with a “serious illness” (mostly cancer).^17^ The topics of their classroom training were similar to ours, although delivered over 1 day instead of three. In another program, “care coordinator assistants,” who were members of a home-based health care team program for patients with dementia were trained to “facilitate ACP discussions.”^13,14^ Their training curriculum comprised four workshops that included training in the use of Go Wish Cards (a card set designed to assist patients and families communicate about values and goals of care), two simulation sessions, and weekly case conferences. Unlike our program, a major component of the care coordinator assistant role was general health care, unrelated to ACP and end-of-life decision making.

The Patient Care Connect Program (PCCP) prepared “lay patient navigators” to “facilitate conversations about ACP” with patients with cancer.^21,23^ To train its navigators, who were linked to 12 cancer health centers in the southeast United States, the PCCP added 1 day of in-person training to the training protocols developed by the Respecting Choices program.^21^ Respecting Choices is a program of six on-line modules focusing on: concepts of communication, advance directives and ACP, selection of health care agents, and health policies relevant to ACP.^40^ The PCCP curriculum contained no experiential, case discussions to support its navigators. Compared with these three programs, our training approach prepares LHWs for more than ACP discussions and provides a more conducive environment (church-based, peer-supported learning) for LHWs to learn how to integrate their spiritual support with their content expertise in ACP and end-of-life decision-making.

Our approach to curriculum development, content, format, and implementation has some limitations. It requires a strong church partnership, which can be labor intensive as is the case for most health delivery partnerships with community-based organizations.^26,34^ The format of the curriculum must be modified to meet the preferences of the church partners; this need should be expected of community-based research and health delivery partnerships. Our print materials and support tools are less extensive than other curricula but can be modified to incorporate other media. The extensive training of up to 200 h for full-time LHWs employed by hospitals or health systems is not feasible for part-time LHWs whose primary link is to a community entity.^13,15^ This limitation is counterbalanced by the enthusiasm of our LHWs for helping (ministering to) fellow congregants. In addition, the church setting provides opportunities for LHWs to serve persons with whom they may already have a trusting relationship. Our LHWs do not function in a system in which they are integrated with health professionals who care for the clients supported by the CCSs. In an integrated system, LHWs are taught to access and insert information in electronic health records, giving them the capacity to communicate with health professionals, thus reducing fragmentation of care and improving quality.^41,42^ However, in some instances, health professionals, who were expected to collaborate with LHWs on ACP, have failed to embrace the role and value of the LHWs and have been reluctant to communicate with them.^22,23^ When this failure occurs, the ostensible benefits of integration are not achieved. A community-based (in this case the church) LHW model is a necessary and important complement to integrated LHW models.^14,43^

## Conclusion

We have demonstrated that a partnership of academia with the African American Church can create a curriculum for training LHWs for the principal role of enhancing and facilitating communications about end-of-life decision-making. Lay health workers who support African Americans with life-limiting illnesses require comprehensive training. Preparing LHWs to facilitate conversations about ACP and advance directives is necessary but insufficient. Lay health workers should also be prepared for the stress of supporting persons and families during the advanced stages of a life-limiting illness and prepared to attend to the spiritual needs of patients or clients. Experiential training and case discussions are essential to clarify concepts, and to maintain the confidence and reduce the anxiety of the LHWs. Our next step is evaluating the impact of the curriculum on the learners and clients.

### Health equity implications

Health inequities of African Americans at the end of life require new interventions and approaches to care. Funders and health systems are increasingly deploying LHWs to meet this need. Communications of LHWs with African Americans about ACP and end-of-life decision-making should be buttressed by community-centered discussions and education.^43^ Our training curriculum responds to this need by describing a church-based approach to educating and training LHWs. This approach complements and responds to the shortcomings of existing curricula.

## Abbreviations Used

ACP: advance care planning
CCSs: comfort care supporters
LHWs: lay health workers
LIGHT: Listening, Identifying, Guiding, Helping, and Translating
PCCP: Patient Care Connect Program
PCH: palliative care and hospice
